# A family of *E. coli *expression vectors for laboratory scale and high throughput soluble protein production

**DOI:** 10.1186/1472-6750-6-12

**Published:** 2006-03-01

**Authors:** Lisa D Cabrita, Weiwen Dai, Stephen P Bottomley

**Affiliations:** 1 Department of Biochemistry and Molecular Biology, School of Biomedical Sciences, Monash University, Clayton, 3800, Australia

## Abstract

**Background:**

In the past few years, both automated and manual high-throughput protein expression and purification has become an accessible means to rapidly screen and produce soluble proteins for structural and functional studies. However, many of the commercial vectors encoding different solubility tags require different cloning and purification steps for each vector, considerably slowing down expression screening. We have developed a set of *E. coli *expression vectors with different solubility tags that allow for parallel cloning from a single PCR product and can be purified using the same protocol.

**Results:**

The set of *E. coli *expression vectors, encode for either a hexa-histidine tag or the three most commonly used solubility tags (GST, MBP, NusA) and all with an N-terminal hexa-histidine sequence. The result is two-fold: the His-tag facilitates purification by immobilised metal affinity chromatography, whilst the fusion domains act primarily as solubility aids during expression, in addition to providing an optional purification step. We have also incorporated a TEV recognition sequence following the solubility tag domain, which allows for highly specific cleavage (using TEV protease) of the fusion protein to yield native protein. These vectors are also designed for ligation-independent cloning and they possess a high-level expressing T7 promoter, which is suitable for auto-induction. To validate our vector system, we have cloned four different genes and also one gene into all four vectors and used small-scale expression and purification techniques. We demonstrate that the vectors are capable of high levels of expression and that efficient screening of new proteins can be readily achieved at the laboratory level.

**Conclusion:**

The result is a set of four rationally designed vectors, which can be used for streamlined cloning, expression and purification of target proteins in the laboratory and have the potential for being adaptable to a high-throughput screening.

## Background

The establishment of rapid expression and purification procedures for recombinant proteins has become a major challenge for biotechnology and indeed any laboratory which studies proteins. The genomic and structural genomic communities have driven the development of high-throughput cloning, expression and purification technologies to a large extent. Recent developments including filter-plate based assays for cloning, expression and purification, ligation-independent cloning (LIC) [1], Gateway technology [2] and auto-induction for protein expression [3] are now readily coupled to robotic pipelines that have made the parallel production of proteins a relatively simple, cost-effective approach. *E. coli *remains the system of first-choice for expressing proteins, as it is cheap and easy to handle, however many mammalian proteins cannot be successfully expressed in *E. coli *[4]. This leaves the researcher to either explore expression space using a range of alternative *E. coli *strains, different temperatures, solubility tags or choose an alternative expression host [5,6].

There are numerous commercial and non-commercial *E. coli *expression vectors available that incorporate fusion tags (for both purification and enhanced solubility), Gateway or LIC technologies, protease cleavage sequences and regulable expression levels [7]. These vectors are generally designed to be used in diverse expression/purification applications and thus often contain extraneous sequences at either the N- or C-termini of the protein. In addition, the use of different fusion tags mean that alternative purification protocols are required for each protein expressed from a different vector, increasing the time it takes to find successful expression and purification conditions. As a result of these limitations, we set out to create a set of *E. coli *expression vectors that incorporate these essential features: i) protein expressed from each vector is initially purified in the same manner; ii) the cloning procedure is the same for all vectors and iii) a range of solubility tags are available. Moreover these vectors have been developed for use at the general laboratory level so that a researcher can rapidly screen a range of expression conditions.

Here, we describe a set of four rationally designed expression vectors for *E. coli*. The first, (pLIC-His) encodes for a hexa-His tag followed by a TEV recognition sequence. The other three encode for three common solubility tags [MBP [8], NusA [9], GST [10] plus a TEV recognition sequence. TEV protease was selected due its superior specificity and adaptability to a range of buffering conditions [11]. The vectors are LIC compatible, and contain an N-terminal hexa-His tag enabling parallel cloning and purification, expression is via a T7 promoter allowing for auto-induction and they all possess a TEV recognition site between the fusion partner and the gene of interest. These features make the vectors ideally suitable for high-throughput cloning and expression screening.

We demonstrate that soluble expression was observed for a range of proteins and that they could be readily digested with TEV to yield the native protein. In addition, we illustrate the relevance of parallel cloning in deciding on a solubility tag that is suitable for a target protein. This demonstrates that the vectors can be used successfully at the laboratory level to rapidly screen target proteins (or their mutants) and can be readily adapted to the high-throughput process.

## Results and discussion

### Construction of the pLIC vectors and His-HHR23A, GST-UIM, MBP-α_1_-AT and Nus-UBL

**Figure 1:**
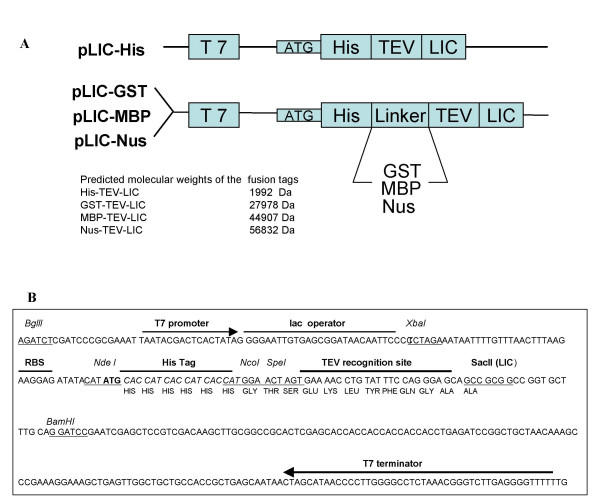
**Basic expression vector design**. (A) The basic vector design for the pLIC vectors. (B) Shows the nucleotide sequence for the features common to all the vectors. The pLIC-His vector is identical in sequence except it does not contain the NcoI-SpeI restriction enzyme sites between the His tag and the TEV recognition sequence.

Several vector systems have been described that incorporate a variety of backbones, promoters, tag positions and cloning procedures. One drawback from several of these studies is the lack of consistency within the vector systems which does not allow a systematic study of protein expression [12-15]. Thus the aim of this study was to generate a set of vectors which enable parallel cloning of a given target(s) and enables a comparative analysis of the expression yields using different solubility tags. As a result, the pLIC set of prokaryotic vectors were created using pET21b(+) as their host. An oligonucleotide cassette was used to introduce the His tag, TEV site and LIC site as well as two unique restriction sites (NcoI-SpeI). These unique sites were used to introduce either the genes encoding for the GST, MBP or Nus tags and could in principle, be used to introduce alternative tags. The four vectors, termed pLIC-His, pLIC-GST, pLIC-MBP and pLIC-Nus were created as shown in Figure 1. It is to our knowledge, the first set of *E. coli *expression vectors derived from the same backbone, with the only variation being in the solubility tag present. The genes HHR23A, UIM, α_1_-AT and UBL were subsequently amplified and successfully introduced into each of the vectors using LIC. In addition, HHR23A was cloned into all vectors simultaneously to monitor the effect that different tags had on the solubility of this particular protein.

**Figure 2:**
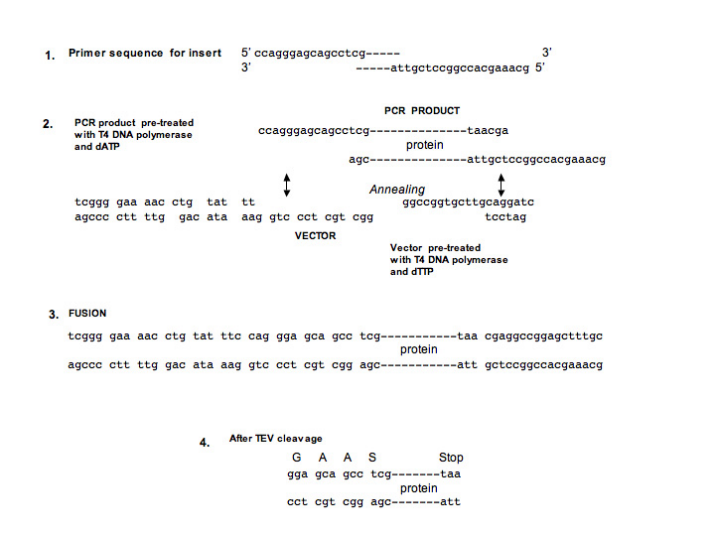
**Ligation-Independent Cloning (LIC) Methodology**. Shows the general procedure for LIC (1) The primer sequences required for amplification of the gene of interest. (2) Both the vector and insert are subject to treatment with T4 DNA polymerase, in the presence of either dTTP (vector) or dATP (insert). (3) The result after successful annealing. (4) After cleavage with TEV protease, the tags are removed and the protein retains 4 amino acids (GAAS).

Two recently developed ligation methods (Gateway technology (Invitrogen) and LIC) have been used extensively by the structural genomics community. Both techniques offer the opportunity for parallel cloning, such that multiple targets can be easily cloned into several different vectors. With the LIC strategy in particular, target genes are amplified with 12–15 bp end sequences and the 5'-3' endonuclease activity of T4 DNA polymerase is then exploited [1,16] (Figure 2). This digestion event generates overhangs in the PCR product that allows subsequent insertion into a complementarily digested vector using a simple annealing step. As there is no need for restriction enzymes or ligation steps, LIC is a highly efficient and cost effective means of cloning. It also has the advantage that it does not require expensive reagents or multiple manipulations.

The LIC technique was found to be an efficient process in our hands, whereby up to 80% of the colonies screened were found to be positive. We observed, however, that success in LIC lies in the quality of the linearized vector, which was dependent, in part, on using a vast excess of SacII enzyme. Gel purification was used in the preparation of the vector and insert and it serves three purposes: by removing unwanted (and possibly active) DNA polymerase, removing excess dNTPs and selecting the correct PCR product over other misprimed events or linearized vector over undigested material. In a high-throughput format, a sizeable quantity of PCR product would be generated (and can be verified by sequencing) prior to parallel cloning into the vectors.

### Expression and purification of the constructs

**Figure 3:**
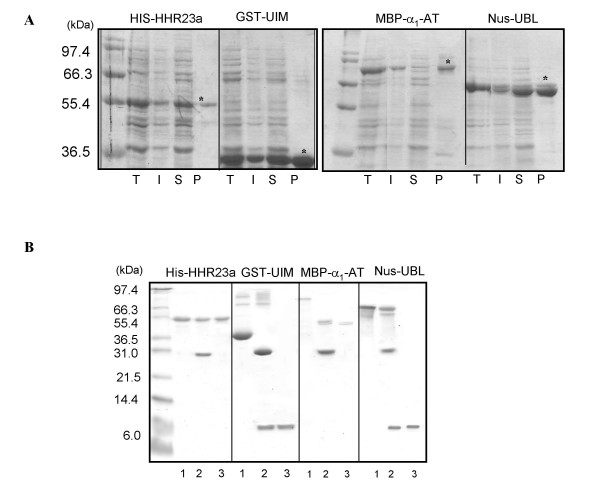
**Small scale expression, purification and cleavage of recombinant proteins**. (A) SDS-PAGE analysis of the total cell lysate (T), insoluble (I), soluble (S), and the purified (P) fractions for each of the fusions: His-HHR23a, GST-UIM, MBP-α_1_-AT and Nus-UBL (the protein is marked with an asterisk). (B) SDS-PAGE analysis of each fusion during TEV protease digestion, (1) intact fusion (2) TEV digestion (3) purified protein after digestion. The band around 30 kDa observed in lane 2 of each experiment corresponds to the TEV protein.

Each of the constructs was transformed into the *E. coli *host BL21(DE3) and expressed in 2 ml of auto-induction expression media. Following expression, the cell density of each of the cultures were normalised to an OD_600 nm _of 5.0 prior to lysis, which allowed for a uniform number of cells between each sample. The cultures were chemically lysed using PopCulture (Novagen), which is a chemical method for lysis and particularly useful for multiple samples. After lysis, the cell lysates were subjected to a solubility assay (as described in the Materials and Methods) to assess the relative proportions of soluble and insoluble expression. As shown in Figure 3A, all of the constructs were found to have soluble expression, as judged by presence of a respective bands at the predicted molecular weights. Both His-HHR23A and MBP-α_1_-AT have marginally greater proportions of insoluble material, whilst Nus-UBL and GST-UIM have slightly elevated proportions of soluble material. Optimization of soluble yields was not performed in this study, as this can vary greatly from protein to protein. It has been recently been shown however, that a lower temperature used during auto-induction may improve the yield of soluble material [3]. The soluble expression and purification of MBP-α_l_-AT was particular interesting, as α_l_-AT is generally produced in very high yields in an insoluble form and purified using a refolding method [17,18].

**Figure 4:**
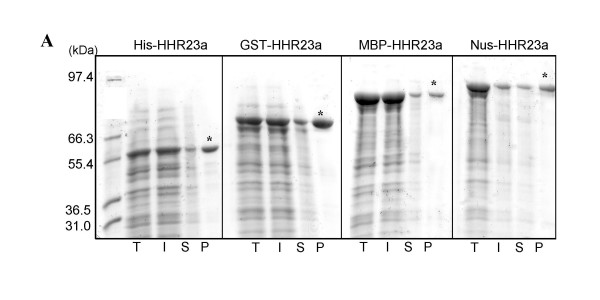
**Parallel expression, purification and cleavage of HHR23A**. HHR23A was cloned into all 4 vectors and the constructs were expressed and purified as described in the materials and methods. SDS-PAGE analysis of the total cell lysate (T), insoluble (I), soluble (S), and the purified (P) fractions for each of the fusions are shown.

In addition to screening different targets in each of the vectors, we also cloned a single gene (HHR23A) into all four vectors. As can be seen in Figure 4, HHR23A expressed largely as inclusion bodies, however soluble material was obtained in all four vectors. More importantly however, is that the tags had differing effects on the expression of HHR23A. In this instance, GST was found to be the most efficient in improving the expression whereas NusA had little additional benefit (Figure 4). This illustrates therefore, the usefulness of parallel cloning and expression, as a protein may express more favourably with a different tag.

An additional observation of the small-scale expression was the importance for a purification step (Ni-NTA). In some cases, soluble expression of the protein showed a very faint band in the soluble fraction, but was purified at more detectable quantities (see α_1_-AT band, Figure 3A). This however, appears to be dependent on the degree of soluble expression, as the UBL and UIM constructs for instance, showed very clear results in the solubility assay (Figure 3A). The use of the solubility assay in isolation should thus be used with caution, particularly for a poorly expressing protein that may produce unclear results. In some cases, it may be useful to complement the assay with a Western blot.

### TEV cleavage

Generally, affinity tags are removed after purification as they can interfere with either the protein's function or with downstream processes. The most common example is if the protein is to be used for crystallographic or NMR studies. While there have been reports of proteins being crystallized with the assistance of an affinity tag [19-21] the general view is that they should be removed [22]. Each of the constructs described above has a TEV cleavage site which can be used to remove the affinity tags, leaving the native protein with 4 vector-derived residues (GAAS) (Figure 1). This is comparable to another LIC vector available which describes 3 vector-derived residues (S-N-A) [14]. Indeed the introduction of non-native amino acids at the N- terminus is not ideal and it is a common problem that generally exists both for the LIC and Gateway technologies. As a result, the effect of such residues (both in number and composition) on the expression, purification and integrity of a given protein must be verified experimentally [12].

To validate the TEV cleavage site, each of the proteins were digested with his-tagged TEV protease and purified. As seen in Figure 3B, after cleavage three species are present: the solubility tag (26 kDa GST, 42 kDa MBP, 54 kDa NusA), the his-tagged TEV protease (30 kDa) and the protein of interest. This mixture can be purified further to remove the his-tagged TEV protease and the solubility tag by selectively binding them to a Ni-NTA column. The protein of interest, on the other hand remains unbound to the Ni resin as it lacks a His tag (Figure 3B).

It is should be noted that although a given protein target may express solubly, its behaviour in the absence of the solubility tag may be very different, as this is a function of other factors (structure, amino acid sequence, aggregation potential, buffering conditions). Thus the integrity of the protein after TEV cleavage should be verified using other methods (dynamic light scattering, size exclusion chromatography, circular dichroism etc.).

## Conclusion

Here, we have presented a set of four rationally designed T7-based *E. coli *expression vectors whose features include the incorporation of solubility tags to assist in expression/purification, a TEV cleavable sequence and a LIC sequence. We have shown soluble expression and purification of four different proteins as well the ability to remove the tags using TEV protease. These vectors allow for rapid parallel cloning and expression and are suitable for either laboratory-scale screening of target proteins, or alternatively for high-throughput screening.

## Methods

### Construction of pLIC-HIS and pLIC-MGN

The vector pET21b(+) (Novagen) was used as the basis for the pLIC vectors. pET21b(+) was manipulated to produce two different constructs, pLIC-His and pLIC-MGN. For the pLIC-HIS construct, the vector's unique NdeI-BamHI restriction sites were used. An oligonucleotide cassette containing the His-tag, TEV and LIC sites was created and ligated into the vector which had been previously digested with NdeI-BamHI (Figure 1). The cassette was comprised of the following oligonucleotides: 5'-tatgcaccatcaccatcaccatgaaaacctgtatttccagggagcagccgcggccggtgctttgcag-3' and 3'-acgtggtagtggtagtggtacttttggacataaaggtccctcgtcggcgccggccacgaaacgtcctag-5'. pLIC-MGN was created by utilising the same NdeI-BamHI sites, but incorporating a cassette of the following oligonucleotides: 5'-tatgcaccatcaccatcaccatggaactagtgaaaacctgtatttccagggag cagccgcggccggtgctttgcag-3' and 3'-acgtggtagtggtagtggtaccttgatcacttttggacataaagg tccctcgtcggcgccggccacgaaacgtcctag-3'. This cassette encodes the His tag, a unique NcoI site, a unique SpeI site, TEV recognition sequence and the LIC site (Figure 1). It was also ligated into pET21b(+).

### Construction of pLIC-GST, pLIC-MBP and pLIC-Nus

The introduction of the unique NcoI and SpeI site in pLIC-MGN allowed for the introduction of the solubility tags: GST, MBP and NusA. The GST gene was cloned from the vector pET41 (Novagen), the MBP gene from pMAL-2cx (New England Biolabs) and NusA from pET43.1 (Novagen). The oligonucleotides used for the amplification are presented in Additional file 1. As the NusA gene already contained a NcoI site, the QuikChange site-directed mutagenesis technique (Stratagene) was used to remove this site, using the vendor's instructions. Briefly, the plasmid was amplified using a pair of complementary primers (See Additional file 1) and using the polymerase *Pfu*. The PCR product was then digested with 10 u of DpnI, followed by transformation into competent DH5α *E. coli*.

The genes encoding GST, MBP and NusA were amplified and each of the PCR products were subjected to sequential digestion with NcoI and SpeI followed by gel purification and ligation into the pLIC-MGN vector. This yielded three different constructs termed: pLIC-GST, pLIC-MBP and pLIC-Nus.

### Cloning of UIM, α_1_-AT, HHR23A and UBL into the vectors

To clone into the expression vectors, PCR products of the gene of interest need to be generated with specific overhangs that are complementary to the sequence of the vector (Figure 2). After cleavage with TEV, the native protein will have four vector-derived residues (G-A-A-S). To validate the vectors, four genes were chosen to be expressed: HHR23A was cloned into pLIC-His, ubiquitin interacting motif (UIM) into pLIC-GST, α_1_-antitrypsin (α_1_-AT) in pLIC-MBP and ubiquitin-like domain from HHR23A (UBL) into pLIC-NUS.

The LIC site consists of a unique SacII site, which can be used to linearize the vector and thus permit T4 DNA polymerase treatment to take place (Figure 2). 30 units of SacII (New England Biolabs) was used to digest 1 μg of the target vector in a total reaction volume of 50 μl for a total of 3 hours. The linearized vector was then separated and gel purified from an agarose gel and the DNA concentration determined using UV-spectroscopy. 0.2 pmol of DNA was then treated with T4 DNA Polymerase (1 u/0.1 pmol DNA) and 2.5 mM dTTP at 22°C for 30 minutes followed by heat inactivation for 20 minutes.

Each of the four genes was amplified using *Pfu *polymerase (Promega) using the oligonucleotides detailed in Additional file 1. The amplification was for 30 cycles with the following conditions: 95°C 1 min, X°C 1 min, 72°C for Ymins. The annealing temperatures (X) and extension times (Y) for each of the constructs are shown in Additional file 1. After amplification, the PCR products were gel purified prior to being treated with T4 DNA Polymerase (1 u/0.1 pmol) and 2.5 mM dATP at 22°C for 30 mins, followed by a 20 min heat inactivation step.

For the annealing process, 1 μl of T4 treated vector was mixed with 2 μl of T4 treated PCR product and allowed to incubate at 22°C for 1 hour. 1 μl of 25 mM EDTA pH 8.0 was then added and the reaction incubated for 5 mins. The annealed mix was then transformed into competent JM107 *E. coli*. The colonies were assessed by colony PCR and the clones were verified using DNA sequencing.

### Expression and purification of His-HHR23A, GST-UIM, MBP-α_1_AT and Nus-UBL

Each of the clones were transformed into competent BL21(DE3) *E. coli *cells and streaked onto plates. A single colony was selected and expressed in 2 ml of Overnight Express Media (Novagen) for 18 hours at 30°C, 300 rpm. The OD_600 nm _was recorded for cells after growth (typically ranging between 8–11) and the absorbance was normalised to OD_600 nm _= 5.0 for each, which allowed for a uniform number of cells between samples. The cells were then lysed with 0.2 ml of PopCulture supplemented with 2 μl of lysozyme and benzoase, as per manufacturer's recommendation (Novagen). 200 μl of each cell lysate was retained for the Solubility Assay (see below). The remaining material was bound batch wise, to 200 μl of Ni-NTA resin which had been previously equilibrated in buffer (25 mM NaPO_4_, 500 mM NaCl pH 8.0). The slurry was then collected in a 2 ml filter plate (supplied with the solubility assay kit, Novagen) and washed with 4 ml of buffer supplemented with 25 mM imidazole. The proteins were then eluted with 250 mM imidazole in 25 mM NaPO_4_, 50 mM NaCl pH 8.0 in a 200 μl volume.

### Solubility assay

The solubility assay (Novagen) was used to determine the relative proportions of soluble and insoluble material that were expressed for each of the constructs. After lysis with PopCulture, 200 μl samples of each lysate was passed through the wells of a 0.2 μm filter plate (supplied by the manufacturer) and collected. Here, the soluble protein passes through the plate whilst the insoluble inclusion bodies are retained. 200 μl of 4%(w/v) SDS was then added to each well and following a 10 min incubation the insoluble proteins eluted. A sample of both the soluble and insoluble fractions were retained for SDS-PAGE analysis.

### TEV protease cleavage

The concentration of protein was estimated using the BCA Assay (Pierce). Protein at a concentration of 0.5 mg/ml was incubated with 0.05 mg/ml of TEV and allowed to incubate at 4°C for 16 hours. The sample was then run on a SDS gel to check for the completion of cleavage

## Authors' contributions

LC carried out the cloning and expression studies, participated in the design of the study and drafted the manuscript. WD participated in the molecular biology. SB conceived of the study, and participated in its design and coordination and helped to draft the manuscript. All authors read and approved the final manuscript.

## Supplementary Material

Additional File 1 Oligonucleotide sequences used to isolate the genes for GST, MBP and Nus, as well as the annealing and extension times used for amplification.
