# Supplementary material for: A family of E. coli expression vectors for laboratory scale and high throughput soluble protein production
Source: BMC Biotechnol. 2006 Mar 1;6:12. doi: 10.1186/1472-6750-6-12 (PMC1420288; doi:10.1186/1472-6750-6-12)
Supplement: Additional File 1 — Oligonucleotide sequences used to isolate the genes for GST, MBP and Nus, as well as the annealing and extension times used for amplification. [file 1472-6750-6-12-S1.doc]

#### Table 1:

Oligonucleotide sequences used to isolate the genes for GST, MBP and Nus, as well as the annealing and extension times used for amplification. In addition the primer sequences used to generate the genes for protein expression are shown. (*) This oligonucleotide was used to delete the Nco1 site in NusA. The mutation is underlined.

| **Gene** | **Forward Oligonucleotide** | **Reverse Oligonucleotide** | **Annealing Temp (ºC)** | **Extension**  **Time**  **(min)** |
| --- | --- | --- | --- | --- |
| MBP | ggcgcgcaccatggaaaaatcgaagaaggt | cgcgccaccactagtgttgttgttatt | 59 | 1.2 |
| GST | ttagctcaccatggatcccctatactaggt | cagaccactagttgaaccatccgattttgg | 57 | 0.75 |
| NusA | ggccgccgccatggaaacaaagaaattttg | agaaccactagtcgcttcgtcaccgaacca | 58 | 1.2 |
| NusAdelNco1 * | gaagataaacacacgatggacatcgcc | ggcgatgtccatcgtgtgtttatcttc | 55 | 13.5 |
| UIM | ccagggagcagcctcgatgttagaagcaaatgatg | gcaaagcaccggcctcgtcagcggagatctgc | 55 | 0.5 |
| UBL | ccagggagcagcctcgatggccgtcaccatc | gcaaagcaccggcctcgtcaggctttggtcttgg | 55 | 0.5 |
| HHR23A | ccagggagcctcgatggccgtcaccatc | gcaaagcaccggcctcgtcactcgtcatcaaa | 55 | 2.5 |
| α_1_-AT | ccagggagcagcctcggatcaggatcaccca | gcaaagcaccggcctcgttatttttgggtggg | 55 | 2.5 |
